# Characterization of oncogenic pathways linked with T cell exclusion in urothelial bladder cancer

**DOI:** 10.1186/2051-1426-3-S2-O14

**Published:** 2015-11-04

**Authors:** Randy F Sweis, Stefani Spranger, Jason J Luke, Thomas F Gajewski

**Affiliations:** 1 University of Chicago, Chicago, IL, USA

## 

Response to immunotherapies and improved survival are linked to a T cell-inflamed tumor microenvironment, which is characterized by expression of a chemokine and type I interferon gene signature and the presence of CD8^+^ tumor-infiltrating T cells. In urothelial bladder cancer, durable responses to PD1-targeted immunotherapies have been reported, yet the majority of patients still do not benefit clinically. To better understand mechanisms of resistance, we profiled somatic mutations unique to non-T cell-inflamed urothelial bladder cancers and identified upregulated molecular pathways. RNA sequencing and exome somatic mutation data from 237 bladder cancers in The Cancer Genome Atlas were analyzed. We first confirmed that 32% of urothelial bladder tumors were non-T cell-inflamed by immune gene expression profiling. This group had reduced expression of immune inhibitory markers PDL1, LAG3, FOXP3, TIM3, IDO1, (*P* < 0.0001, Wilcoxon test for each) and an absence of CD8^+^ tumor-infiltrating T cells (≤1 per 40x field) by immunohistochemistry performed on available samples (*P* = 0.01, Fisher's exact test). Other samples were classified as either T cell-inflamed (36%) or intermediate (32%). We detected 56,052 non-synonymous mutations in all samples affecting 9,717 genes. To investigate the relationship between overall mutational burden and T cell-inflamed/non-T cell-inflamed tumors, we calculated the total number of mutations (MAF < 0.01) per sample by group. We found no difference in mutational density between groups (*P* = 0.80, two-sided t-test). We next evaluated whether mutations in specific genes could be linked with T cell exclusion. Of the genes mutated in at least five samples, 24 occurred only in non-T cell-inflamed tumors (*P* < 0.05, Fisher's exact test). The most common of those genes was FGFR3, which was mutated in 11 non-T cell-inflamed samples. Lastly, we applied Ingenuity Pathway Analysis to the subset of genes significantly upregulated in the non-T cell-inflamed tumors and found Wnt/β-catenin and peroxisome proliferator-activated receptor gamma (PPARG) to be the most active (*P* = 0.003 for each). In summary, we outline the first characterization of urothelial bladder cancer subtypes by immune gene expression profiling, and find that mutational burden does not vary between them. However, specific gene mutations and molecular oncogenic pathways show a strong correlation with the non-T cell-inflamed urothelial bladder subtype.

